# Knowledge and trust of mothers regarding childhood vaccination in Rwanda

**DOI:** 10.1186/s12889-024-18547-1

**Published:** 2024-04-17

**Authors:** Edward Mbonigaba, Fengyun Yu, Mark Donald C Reñosa, Frederick Nchang Cho, Qiushi Chen, Claudia M Denkinger, Shannon A McMahon, Simiao Chen

**Affiliations:** 1 Centre of Infectious Diseases, Division of Infectious Disease and Tropical Medicine, Universität Heidelberg, Heidelberg, Germany; 2 College of Medicine and Health Sciences, School of Public, Health- University of Rwanda, Kigali, Rwanda; 3 Interdisciplinary Centre for Scientific Computing, Universität Heidelberg, Heidelberg, Germany; 4 Heidelberg Institute of Global Health, Universität Heidelberg, Heidelberg, Germany; 5 Department of Epidemiology and Biostatistics, Research Institute for Tropical Medicine, Muntinlupa City, Philippines; 6 Buea, Cameroon; 7 Infectious Disease Laboratory, Faculty of Health Sciences, University of Buea, Buea, Cameroon; 8 Department of Industrial and Manufacturing Engineering, The Harold and Inge Marcus, The Pennsylvania State University, University ParkHarrisburg, PA USA; 9 Chinese Academy of Medical Sciences and Peking Union Medical College, Peking, China

**Keywords:** Childhood vaccination, Assessment, Knowledge, Trust, Rwanda

## Abstract

**Introduction:**

Knowledge and trust are some of the contributing factors to vaccine acceptance(VA) and Vaccine hesitancy (VH) is one of the top threats to global health. A significant drop in childhood vaccination has been observed in recent years. One important reason that influences mothers' choice to either postpone or avoid children's vaccinations is knowledge and trust in childhood vaccines. This study aimed to assess mothers' knowledge and trust on vaccination of their children, and to examine the association between vaccination knowledge and selected socio-demographic factors.

**Methods:**

A cross-sectional survey was conducted from January 2022 to March 2022 to assess the knowledge and trust of mothers regarding childhood vaccination. Data was collected with self-administered questionnaires. Multivariable logistic regression analysis was employed to assess factors associated with childhood vaccine knowledge and trust.

**Results:**

Of the 2,126 Rwandan parents who participated in the study, the proportions with good knowledge of – and good trust in childhood vaccination were 95.5% and 91.4%, respectively. The popular sources of information about childhood vaccination were health care professionals (91.8%) and mass media (28.9%). Multinomial logistic regression analysis showed that good knowledge of – and trust in childhood vaccination were associated with the relationship with child(ren), education, occupation, and monthly income. The Multinomial logistic regression also revealed that the determinants of good knowledge of – and trust in childhood vaccination were; caregiver (*p* = 4.0 × 10^–4^, adjusted Odds Ratio (aOR); 1.7, 95%C.I; 1.3 – 2.3), no formal educational status (*p* = 3.3 × 10^–2^, aOR; 1.7, 95%C.I; 1.0 – 3.0), the unemployed occupational status (*p* = 2.4 × 10^–2^, aOR; 1.2, 95%C.I; 1.0 – 1.4), and persons on more than $401 per month (*p* = 2.0 × 10^–4^, aOR; 3.5, 95%C.I; 1.8 – 6.8).

**Conclusion:**

The majority of parents in Rwanda had both good knowledge of—and good trust regarding childhood vaccination. Public health strategies to promote vaccination, education programmes as well as improved communication tools between health care professionals/traditional leaders/religious leaders and parents need to be considered to achieve favourable vaccination attitudes and practices for all parents in Rwanda.

**Supplementary Information:**

The online version contains supplementary material available at 10.1186/s12889-024-18547-1.

## Introduction

Knowledge and trust regarding childhood vaccination influence vaccine uptake in combination with other factors such as vaccine hesitancy (VH). Public health experts broadly agree that vaccinations are essential because they have significantly decreased childhood illness and mortality, particularly by eradicating smallpox worldwide [1, 2]. Yearly, it is believed that immunisation rescue between two and three million people worldwide [1, 3, 4]. Vaccination has significant psychological and developmental implications in contrast to its life-saving and healthcare roles [5–7]. The concept of vaccination is not limited to one person but concerns the entire; childhood vaccination does not only protect children, rather it protects the community by preventing the transmission of vaccine -preventable diseases (VPD) in herd immunity [1, 8]. According to research, when therapy expenses are considered across each penny spent on vaccinations in Africa between 2010 and 2020 will provide a 16-fold yield, considering treatment costs and productivity losses [9]. Religious and traditional disparities have also contributed to increasing, mistrust, and misinformation [10–13], while healthcare personnel especially paediatricians are a reliable source of information on vaccination for parents and their children [14, 15]. However, notwithstanding well-established facts of vaccination's efficacy, public mistrust as well as the lack of confidence in vaccines has been growing for a long time [16–18], and needs to be addressed. The World Health Organisation (WHO) identified VH as one of the most serious health implications to humankind in 2019 and defined it as the referral, deferral, or denial of vaccines regardless of their accessibility [19–21].

Additionally cited as a concern to pandemic preparedness is vaccine VH [22, 23]. VH is the delay in acceptance or refusal of vaccines despite the availability of vaccination services [17, 20, 24]; consequently leading to poor vaccine coverage [25]. Many researchers have connected VH to differing perspectives, insufficient knowledge and trust about vaccinations, trust of the vaccines, trust of physicians and health provider’s advice [1], conveniences, cost of vaccines [26], and perceptions of the threats or advantages of vaccination [27–31]. Although VH is regarded as a worldwide problem, the assessment methods currently in use are inadequate to accurately measure and comprehend the scope to which it has interfered with vaccination programmes in various configurations. In 2020, some studies reported that vaccine trust varies among and within states, and that low confidence negatively impacts low- and middle-income countries with already fragile healthcare systems [32, 33].

Vaccine acceptance (VA) varies across countries, generations, and the personality of individuals [34, 35]. Various factors affect VA; good knowledge of the vaccination process, educational status, employment status, the safeness and effectiveness of vaccines [14, 15, 34]. Factors associated with VA or VH could be individualistic and include risk perceptions, as well as (dis)trust [35].

In East Africa, complete basic childhood vaccine coverage remains a major public health concern with significant differences across countries. It was revealed that complete basic childhood vaccination was significantly associated with parental education and media exposure [36], which is synonymous to knowledge of childhood vaccination. Research findings also indicate that knowledge about, and attitude towards, childhood vaccination among mothers in Saudi Arabia and Cyprus are excellent [6, 36, 37].

Furthermore, additional research is necessary to identify the environmental and specific variables that raise the risk of VH and to learn more about efficient ways to boost vaccine uptake [38–40].

Throughout the 1994 genocide against the Tutsis in Rwanda, the country's immunisation rate was significantly under 30%, and a high prevalence of infections that could have been prevented by vaccination was observed; for instance, 28,000 measles cases were reported in 1995 [38, 39]. Regarding that, the Rwandan government, with the support of the World Health Organisation [41] made significant investments in public health, and a strong Expanded Programme on Immunisation (EPI) helped to significantly lower childhood hesitancy as well as levels of childhood mortality and morbidity [42–44], with about 28.2% neonate mortality in 2018 and 29.4 deaths per 1,000 live births in 2023 [45, 46]. The acceptance of vaccines is an important predictor of vaccine uptake. This has public health implications as those who are not vaccinated are at a higher risk of infection from vaccine preventable diseases [47]. The responsibility of the EPI is to offer routine vaccination, supplemental immunisation actions, and monitoring systems for vaccine preventable diseases (VPDs) benchmarks. In order to promote immunisation and reduce dropout rates, the EPI works with community health professionals and other well-functioning networks across many sectors in the country [3, 38]. To the researchers' knowledge, little or no studies have been carried out to evaluate the variation in tendencies for immunisation and vaccine effectiveness in Rwanda's health districts. Assessing childhood vaccination rates and knowledge regarding routine immunisation against diseases that can be prevented by vaccines is therefore necessary. To better understand the concept of vaccine uptake, we need to explore factors such a knowledge and trust and how they influence mother perception toward routine vaccines. Low levels of knowledge and trust can harm routine immunization practices in the past as referenced in the discussion Chapter. To this end, the current study aims to quantitatively assess parental knowledge and trust regarding recommended childhood vaccination.

## Materials and methods

### Study framework

Rwanda is a landlocked African country in the Great Lakes region, between 1°04′ and 2°51′ south latitude, and between 28°45′ and 31°15′ East longitude [48]; geographically dominated by mountains in the west and savannah to the east, with numerous lakes throughout the country. The population of Rwanda is 13.46 million (2021) million people, with a growth rate of 2.58% from 2019 to 2020 [49].

### Study design

A national cross-sectional study was conducted from January 2022 to March 2022 among parents selected in five Districts of Rwanda. Our study framework was composed of districts of which two of the five Districts (i.e Nyagatare and Ngoma), are located in the Eastern Province, while Nyamagabe, Nyarugenge, and Ngororero are located in the Southern, Central and Western Provinces respectively.

### Sampling

We used a multistage Cluster (Province) sampling method where a list of all Cells/"Akagalis" and villages therein was drawn. A total of 50 villages were selected, including at least two villages from each Cell. The sampling procedure for the required number of Parents (mothers or caregivers) was done in three stages.

Firstly, four of the five Provinces in Rwanda; the Eastern, Southern, Central, and Western Provinces were selected by simple random sampling using a random sample generator (RSG).

**Fig. 1:**
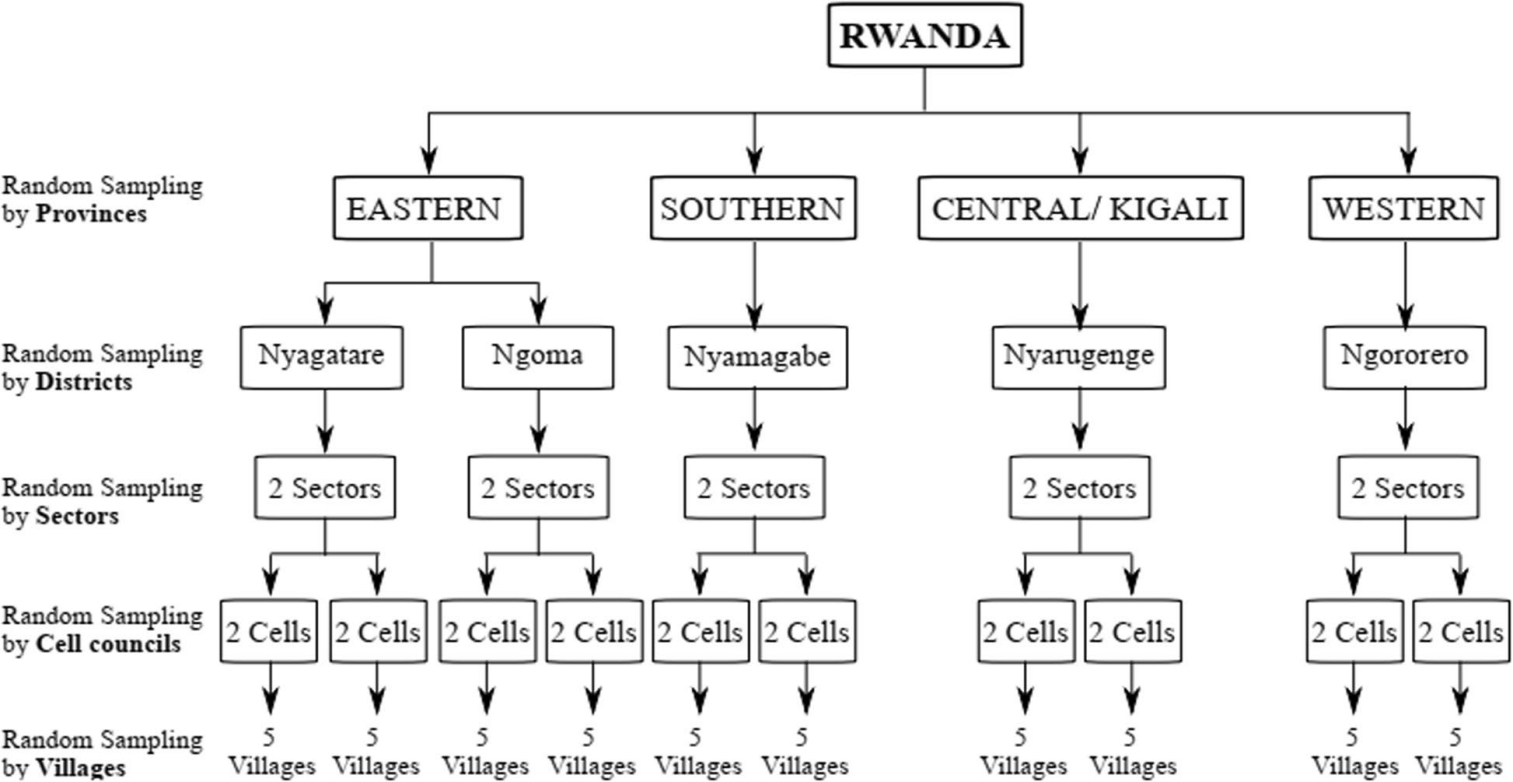
Schematic illustration of the study design and settings; multistage sampling

Secondly, the number of Districts of the selected Provinces were listed and one each was selected using the RSG. Within each District, two Sectors were selected and within each Sector, two Cells/"Akagalis" were selected. At least two villages were then selected from each Akagali by RSG (Fig. 1).

Thirdly, within each selected village, parents were sampled consecutively until the desired sample size was attained. The  conveniet sampling technique was applied wherein participants were approached and informed of the study objectives at their work places and at their doorsteps. We applied conveniet sampling techenique because this study was conducted during the period of COVID-19 where we though reaching our desired sample size would not be reached. Data was then collected through personal interviews with the parents. Despite the non-probabilistic sampling approach used for sampling mothers, we managed to recruit participants from all major areas of the randomly selected villages and from different age and socio-economic strata, thus ensuring a representative sample of the adult female Rwandan population.

Prior to the sampling, research assistants were trained on the objectives of the study, how to administer the data collection tool, how to probe study participants, and how to record responses. Five data quality control officers; one for each district, were also trained on the various types of data collected by the questionnaire, and the use of Microsoft excel.

### Study population and target sample size

The study population constituted parents of children (mothers or caregivers) aged 12 to 23 months, who were conveniently sampled from 50 villages in Rwanda. This convenient sampling approach of mothers was inevitable because all mothers could neither be at home nor at market nor office at the same time. Being a mother or caregiver, ≥ 18 years of age, and granting consent to participate in the study constituted the inclusion criteria. The exclusion criteria from the study were those who had one child(ren) older than five years, not living in the selected Districts and refused to grant consent.

In the absence of similar studies in Rwanda, a minimum sample size of 154 per Cluster/District, based on the WHO immunisation coverage cluster survey [50], was calculated with the CDC Epi Info 7.2.5.0 (Centre for Disease Control, Georgia, USA) StatCalc with the following characteristics: an estimated District population size of 362,806 in 2022 [49], an estimated proportion of mothers with knowledge of – trust in childhood vaccination of 50.0%, a design effect of 2.0, an accepted error margin of 5% [51], and five Clusters/Districts. Assuming and Considering respondents possible non-response and non-responding respondents, the sample size was adjusted by 10% (16 respondents) to 170. We also assumed that some participants would not consent to our study and hence would not be included.

### Definition of concepts and study variables

*Independent/demographic variables*: included age, sex, marital status, relationship with the child(ren), religion, level of education, occupation, number of children in the immunisation bracket, and monthly income.

*Dependent variables*: included sources of information about childhood vaccination (Question 9; S1 Appendix), parental knowledge of childhood vaccination, trust in childhood vaccination, and both parental knowledge of – and trust in childhood vaccination.

Parental trust in childhood vaccination was assessed based on two questions (Questions 27 & 28; S1 Appendix): Do you trust the information you receive about the vaccination of your children? How much do you trust the government and public health agencies in the promotion of childhood vaccines? The potential responses 'yes,' 'no,' and 'I do not know' were scored as one for 'yes', and zero for 'no/I don't know'. Respondents were said to have trust in childhood vaccination if they answered "yes" to both questions; those who answered "no" to one or both questions were considered to have no trust in childhood vaccination, while respondents who answered "I do not know" to one or both questions were considered that they are not sure about their trust in childhood vaccination.

Parental knowledge of childhood vaccination was assessed based on responses to four questions; (Questions 30 – 33, 36; S1 Appendix). The potential responses to each of these questions were sorted on a Likert scale [52] and included strongly disagree, disagree, undecided, agree, and strongly agree. These potential responses were then scored as one, two, three, four, and five, respectively, for questions 30 – 33.

Parental knowledge of – and Trust in childhood vaccination was assessed based on six questions; (Questions 27 & 28; and 30 – 33; S1 Appendix). Modified Bloom's cut-off points were used to rate knowledge of -, trust in -, as well as knowledge of – and trust in childhood vaccination as very poor (< 20%), poor (≥ 20 but < 40%), moderate (≥ 40 but < 60%), good (≥ 60 but < 80%), or very good (≥ 80%) and later as poor (< 60%) or good (≥ 60%) [53, 54].

### Data collection and analysis

Data was collected with the use of well-structured questionnaires in a face-to-face interview. The questionnaire which consisted of 62 questions, aimed to collect information on respondent's identification, demographic characteristics, information about child(ren) immunisation status, and others. The survey instrument took approximately 15–30 min to administer. Prior to the study, the validity of the questionnaire was confirmed by pre-testing in 10 participants who were excluded from the study. Based on the pre-test study, the format and wording of some questions were corrected and refined. Data from the 10 participants was used to assess internal consistency reliability using Cronbach's alpha (α) [55–57]. The results showed adequate internal consistency reliability (with Cronbach's α = 0.72) [56, 57] for the eight sections with 62 questions. At the end of each day or after every two days, the data quality control officers checked the entry of all data into the Microsoft Office Excel sheet to ensure that the right data is being collected. At the end of the data collection exercise, the field supervisor checked all the data from the various districts to ensure that the data collected was in order.

Age groups, sex, marital status, relationship with the child(ren), religion, education, and occupation were summarised as counts and percentages. Age, number of children in immunisation bracket, knowledge of childhood vaccination, and trust in childhood vaccination scores were expressed as ranges and means. Data was entered into a Microsoft Office Excel spreadsheet, double-checked for consistency, exported to, and analysed with CDC Epi Info 7.2.5.0 (Centre for Disease Control, Georgia, USA). Binomial logistic regression analysis as well as multinomial logistic regression (MNLR) were used to determine associations between knowledge of – trust in childhood vaccination, as well as knowledge of – and trust in childhood vaccination with demographic characteristics. Associations between respondent's characteristics (covariates) and Districts were evaluated using the Pearson Chi square (*χ*^*2*^) test. Multicollinearity was tested for, and the following models were used:

Knowledge of childhood vaccination = β_0_ + β_1_Age + β_2_Number of children in the immunisation bracket + β_3_Sex + β_4_Relationship with Child(ren) + β_5_Religion + β_6_Marrital Status + β_7_Education + β_8_Occupation + ε,

Trust in childhood vaccination = β_0_ + β_1_Age + β_2_Number of children in the immunisation bracket + β_3_Sex + β_4_Relationship with Child(ren) + β_5_Religion + β_6_Marrital Status + β_7_Education + β_8_Occupation + β_9_Monthly Income + β_10_Knowledge of Childhood Vaccination + ε, and.

Knowledge of – and Trust in childhood vaccination = β_0_ + β_1_Age + β_2_Number of children in the immunisation bracket + β_3_Sex + β_4_Relationship with Child(ren) + β_5_Religion + β_6_Marrital Status + β_7_Education + β_8_Occupation + β_9_Monthly Income + ε.

Where β_0_ is a constant, β_1_, β_2_, β_3_, β_4_, β_5_, β_6_, β_7_, β_8_, β_9_, and β_10_ are coefficients and ε is the regression error. For multicollinearity, variance inflation factor between 1 and 5 indicated moderate correlation between a given predictor variable and other predictor variables in the model. The significance level was set at 0.05.

### Ethical considerations

This study was conducted in accordance with the Helsinki Declaration [58] and cleared by the Institutional Review Board (IRB) of the University of Rwanda, College of Medicine and Health Science (No. 402/CMHS IRB/2020) and the Ethics Committee of the University of Heidelberg ethical committee (S-829/2021). All participants signed the informed consent prior to being interviewed. All participants were informed and assured that the data collected would be used only for research purposes and their individual responses would not be available to the public.

## Results

### Characteristics of study population

A total of 2,126 respondents were included in this analysis: 456 (21.4%) from Ngoma, 390 (18.3%) from Ngororero, 494 (23.2%) from Nyagatare, 313 (14.7%) from Nyamagabe, and 473 (22.2%) from Nyarugenge (Supplementary File 1).

About half (49.4%, 95%C.I; 47.3 – 51.5) of the respondents were 30 years or less [mean age of 31.03 years (SD 7.5, range 18 – 58)], with less than a tenth (7.3%, 95%C.I; 6.3 – 8.5) who are more than 41 years old, a gross majority; about four-fifth (82.2%, 95%C.I; 80.5 – 83.8) were females, about half (51.4%, 95%C.I; 49.3 – 53.5) had completed the primary level of education, about three-quarters (78.5%, 95%C.I; 76.7 – 80.2) earned less than 100 United States Dollar (USD) per month, close to two-thirds (63.5%, 95%C.I; 61.5 – 65.6) were artisanal workers, and (78.7%, 95%C.I; 76.9 – 80.4) were married (Table 1). Among the 2,126 mothers of the study, 452 (21.3%, 95%C.I; 19.6—23.1) were not married, while 79 (3.7%, 95%C.I; 3.0—4.6) harboured two or more children [mean number of children in the immunisation bracket of 1.04 child (SD 0.24, range 01 – 04)].

**Table 1:** Socio-demographic characteristics of study participants (*n* = 2,126)

General characteristic	Subclass	Count (%)	95% C.I
**Age groups (in years)**	≤ 30	1,050 (49.4)	47.3—51.5
	31 – 40	920 (43.3)	41.2—45.4
	> 41	156 (7.3)	6.3—8.5
	Mean age ($$\overline{x}$$ ± SD)	31.03 ± 6.58	
**Sex**	Male	378 (17.8)	16.2—19.5
	Female	1,748 (82.2)	80.5—83.8
**Relation to child**	Mother	1,805 (84.9)	83.3—86.4
	Caregiver	321 (15.1)	13.6—16.7
**Religion**	Catholic	81 (3.8)	3.1—4.7
	Protestant	2,045 (96.2)	95.3—96.9
	Not married	452 (21.3)	19.6—23.1
	Married	1,674 (78.7)	76.9—80.4
**Education**	No Formal Education	241 (11.3)	10.1—12.7
	Primary	1,093 (51.4)	49.3—53.5
	Secondary	719 (33.8)	31.8—35.9
	Tertiary	73 (3.4)	2.7—4.3
**Occupation**	Artisan	1,351 (63.5)	61.5—65.6
	Casual labour	209 (9.8)	8.6—11.2
	Civil Servant	125 (5.9)	5.0—6.9
	Unemployed	441 (20.7)	19.1—22.5
**# of Children in immunisation bracket**	01	2,047 (96.3)	95.4—97.0
	≥ 02	79 (3.7)	3.0—4.6
	Mean children ($$\overline{x}$$ ± SD)	1.04 ± 0.24	
**Monthly Income ($)**	< 100	1,669 (78.5)	76.7—80.2
	101–200	197 (9.3)	8.1—10.6
	201–300	132 (6.2)	5.3—7.3
	301–400	81 (3.8)	3.1—4.7
	> 400	47 (2.2)	1.7—2.9

1$ = 1.161 RWF = 0.912 € [59], SD: Standard Deviation, number, %; proportion of respondents, 95% C.I; 95% Confidence interval, SD; Standard Deviation

All characteristics of the study participants were significantly associated with districts (Supplementary File 1).

### Sources of vaccine information

**Fig. 2:**
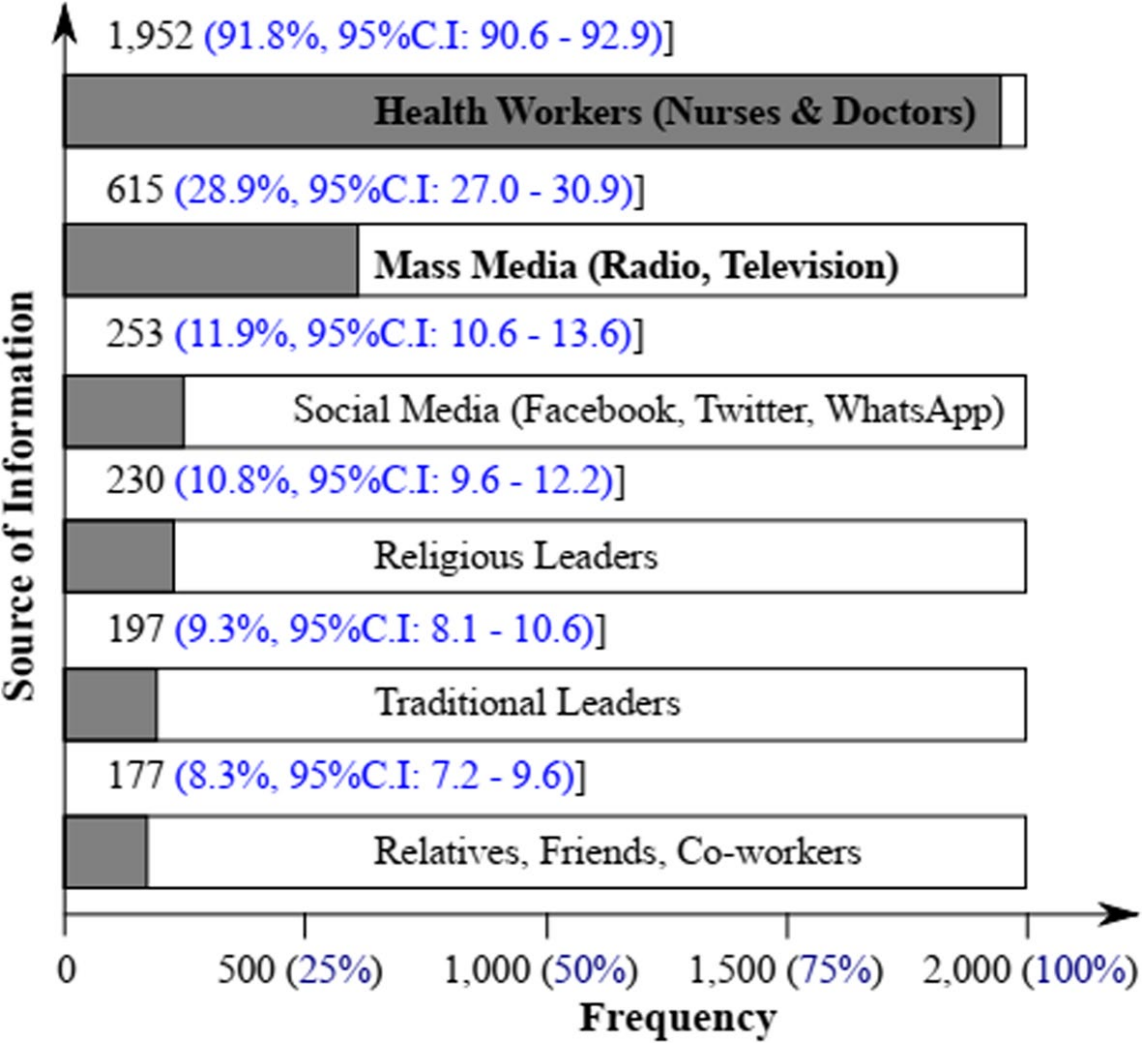
Sources of child vaccination information

Population sources of vaccine information were enumerated as shown in Fig. 2. Most respondents 1,952 (91.8%), had vaccine information from Health Care Workers (Medical Doctors and Nurses), while only a few admitted getting information from Religious (10.8%) and Traditional (9.3%) Leaders. The mass (radio, television, newspapers) and social media (WhatsApp, Facebook, Twitter), as well as relatives, were also sources of vaccine information to the community. Thus, trusted messengers of vaccine information were Healthcare Workers.

### Knowledge and trust of mothers/caregivers regarding childhood vaccination

Mothers and caregivers expressed different shades of knowledge regarding childhood vaccinations. On a five-point Likert scale, 1,136 (53.4%, 95%C.I; 51.3 – 55.6) of the respondents disagreed on the importance of vaccines to their health; 986 (46.4%, 95%C.I; 44.3 – 48.5) strongly agreed on the effectiveness of vaccines; 1,048 (49.3%, 95%C.I; 47.2 – 51.4) also strongly agreed that all vaccines are beneficial to the community; and 1,442 (67.8%, 95%C.I; 65.8 – 69.8) agreed that getting vaccinated is a good way of protecting oneself from vaccine preventable disease (Table 2).

**Table 2:** Knowledge regarding childhood vaccination

	** Question**	** Response**	**Count (%)**	**95% C.I**
**Q30**	Vaccines are important for my health	Strongly Agree	8 (0.4)	0.2—0.7
		Agree	29 (1.4)	0.9—2.0
		Undecided	2 (0.1)	0.0 – 0.3
		Disagree	1,136 (53.4)	51.3 – 55.5
		Strongly Disagree	951 (44.7)	42.6 – 46.9
**Q31**	Vaccines are effective	Strongly Agree	986 (46.4)	44.3—48.5
		Agree	969 (45.6)	43.5—47.7
		Undecided	5 (0.2)	0.1—0.6
		Disagree	123 (5.8)	4.9—6.9
		Strongly Disagree	43 (2.0)	1.5—2.7
**Q32**	Getting myself vaccinated is important for the health of others in my community	Strongly Agree	802 (37.7)	35.7—39.8
		Agree	1,255 (59.0)	56.9—61.1
		Undecided	17 (0.8)	0.5—1.3
		Disagree	27 (1.3)	0.9—1.8
		Strongly Disagree	25 (1.2)	0.8—1.7
**Q33**	All vaccines are beneficial to the community	Strongly Agree	1,048 (49.3)	47.2—51.4
		Agree	1,032 (48.5)	46.4—50.7
		Undecided	16 (0.8)	0.5—1.2
		Disagree	16 (0.8)	0.5—1.2
		Strongly Disagree	14 (0.7)	0.4—1.1
**Q36**	Getting vaccinated is a good way to protect myself from disease	Strongly Agree	607 (28.6)	26.7—30.5
		Agree	1,442 (67.8)	65.8 – 69.8
		Undecided	28 (1.3)	0.9—1.9
		Disagree	25 (1.2)	0.8—1.7
		Strongly Disagree	24 (1.1)	0.8—1.7

Majority [1,255 (59%, 95% C.I; 56.9 – 61.1)] of the respondents agreed on the fact that getting one's self vaccinated, is important for the health of others in the community; thus contributing to herd immunity (Table 2).

**Fig. 3:**
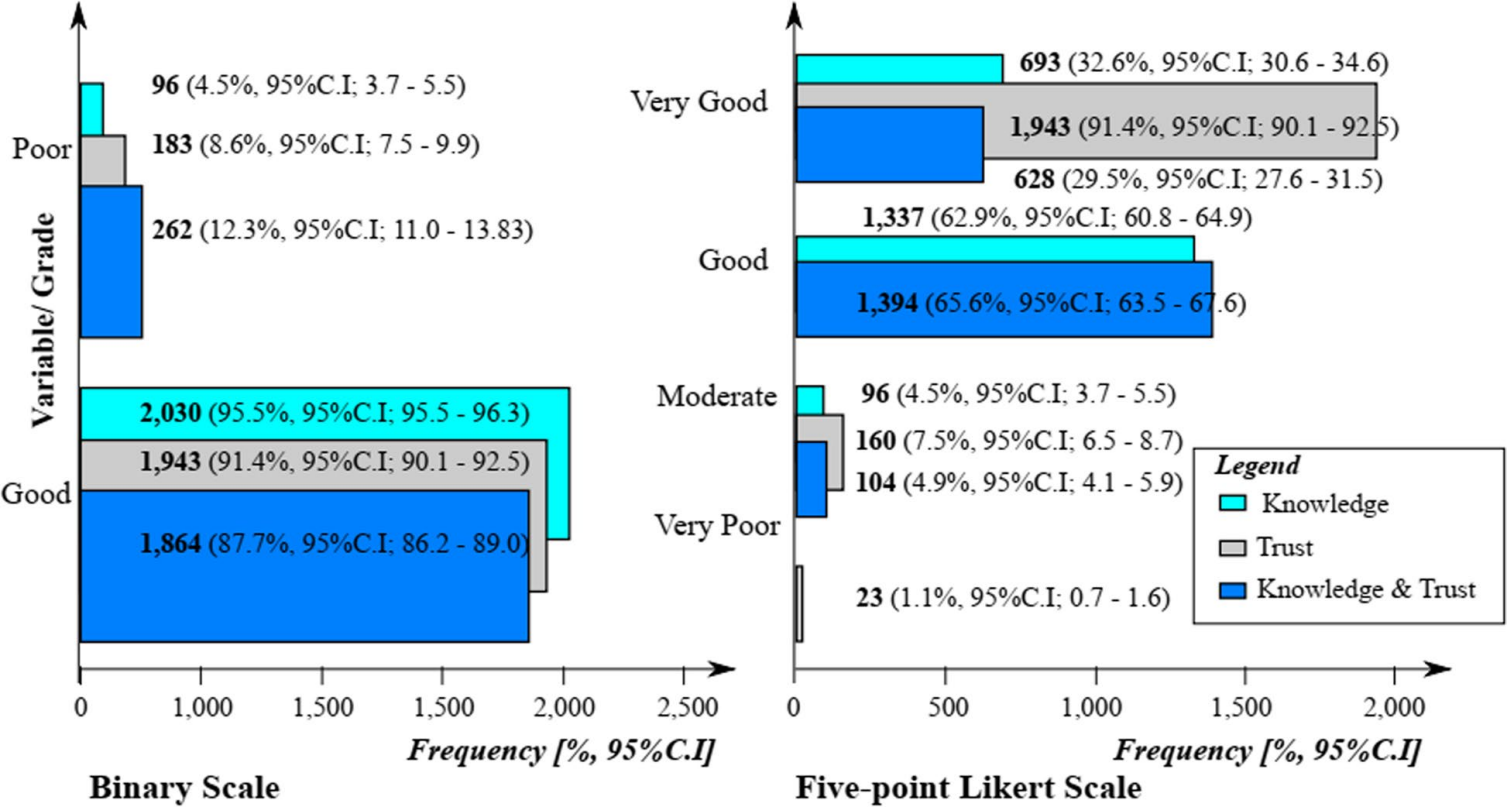
Grading of knowledge, trust, and knowledge/trust

**Fig. 4:**
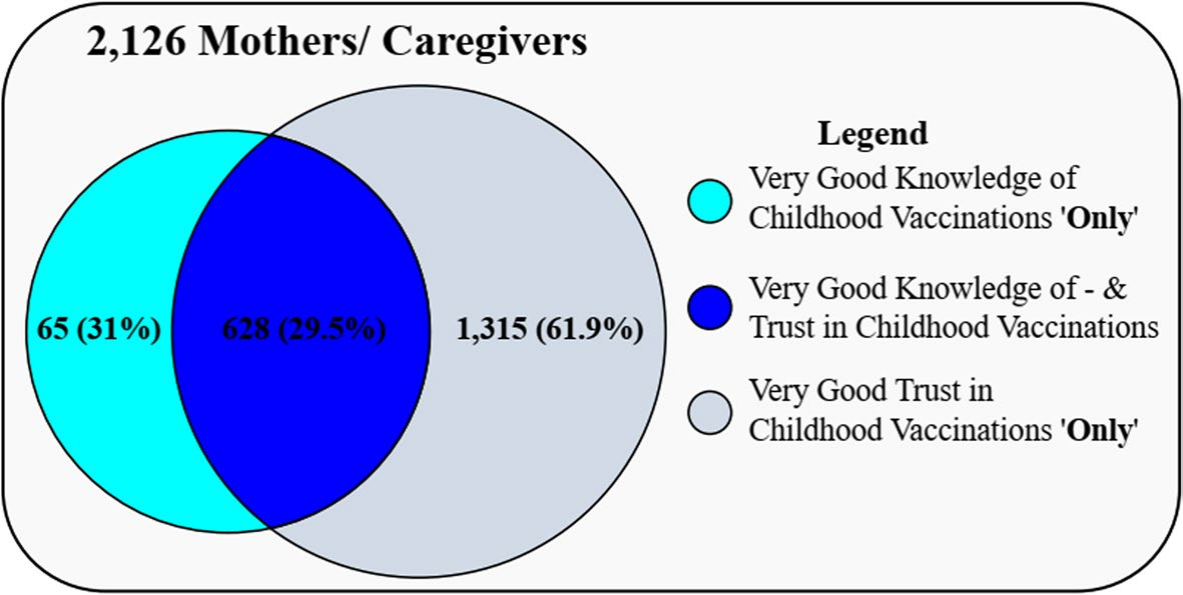
Very Good Knowledge, Trust and both Knowledge & Trust

An absolute majority [1,943 (91.4%, 95% C.I; 90.1 – 92.5)] of the respondents were found to have very good trust in childhood vaccinations, while 2,030 (95.5%, 95% C.I; 30.6 – 34.6) had good knowledge of childhood vaccinations (Fig. 3). The mean knowledge score was 73.18% (SD 7.36, range 40 – 100%), with a median score of 75%. Of the 1,943 mothers/caregivers with very good trust in childhood vaccination, 628 (29.5%, 95%C.I; 27.6 – 31.5) had both very good knowledge of – and very good trust in childhood vaccinations (Figs. 3 and 4).

Binomial logistic regression analysis revealed significant associations of sex, relationship to child(ren), educational status, occupation, and monthly income with good knowledge of childhood vaccinations (*p* < 0.05). From multinomial regression analysis, the odds for having good knowledge of childhood vaccination was higher amongst male respondents (*p* = 1.2 × 10^–2^, aOR; 2.4, 95%C.I; 1.2 – 4.8), respondents who were unmarried (*p* = 3.6 × 10^–3^, aOR; 1.6, 95%C.I; 1.2 – 2.2), and the unemployed (*p* = 1.0 × 10^–4^, aOR; 4.0, 95%C.I; 2.7 – 5.9), those with casual labour (*p* = 1.0 × 10^–4^, aOR; 3.4, 95%C.I; 1.8 – 6.2) as well as civil servants (*p* = 2.5 × 10^–2^, aOR; 2.7, 95%C.I; 1.1 – 6.5) when compared with their various counterparts (Table 3).

**Table 3:** Binomial and multinomial logistic regression analyses of knowledge of childhood vaccination with respondents' characteristics (*n* = 2,030)

		** Binomial regression**			**Multinomial Regression**		
**S/N**	**Characteristic**	***n* (%)**	***χ***^***2***^	***p* – value**	**OR (95% C.I)**	**aOR (95% C.I)**	***p*—value**
**1**	**Age (in years)**						
	≤ 30 (Ref)	998 (49.2)	1.249	5.3 × 10^–1^	1.0	1.0	Ref
	31 – 40	881 (43.4)			*1.2 (0.7 – 1.8)	*1.2 (0.9 – 1.5)	1.0 × 10^–1^
	≥ 41	151 (7.4)			*1.4 (0.5 – 3.7)	*1.6 (0.8 – 2.9)	1.3 × 10^–1^
**2**	**# of Children in immunisation bracket**						
	01 child (Ref)	1,953 (96.2)	0.347	6.0 × 10^–1^	1.0	1.0	Ref
	≥ 02 Children	77 (3.8)			*1.7 (0.4 – 7.4)	*2.4 (0.8 – 6.5)	9.0 × 10^–2^
**3**	**Sex**						
	Female (Ref)	1,659 (81.7)	6.833	**9.0 × 10**^**–3**^	1.0	1.0	Ref
	Male	371 (18.3)			*1.7 (0.6 – 4.6)	*2.4 (1.2 – 4.8)	**1.2 × 10**^**–2**^
**4**	**Relationship**						
	Mother (Ref)	1,714 (84.4)	6.885	**9.0 × 10**^**–3**^	1.0	1.0	Ref
	Caregiver	316 (15.6)			*2.1 (0.6 – 6.8)	*1.8 (0.9 – 3.8)	1.0 × 10^–1^
**5**	**Religion**						
	Catholic	77 (3.8)	0.035	9.0 × 10^–1^	0.7 (0.2 – 1.9)	0.6 (0.3 – 0.9)	**4.3 × 10**^**–2**^
	Protestant (Ref)	1,953 (96.2)			1.0	1.0	Ref
**6**	**Marital status**						
	Not married	435 (21.4)	0.552	5.0 × 10^–1^	*1.4 (0.8 – 2.5)	*1.6 (1.2 – 2.2)	**3.6 × 10**^**–3**^
	Married (Ref)	1,595 (78.6)			1.0	1.0	Ref
**7**	**Educational status**						
	Tertiary (Ref)	72 (3.6)	7.666	**5.0 × 10**^**–2**^	1.0	1.0	Ref
	Secondary	694 (34.2)			0.5 (0.05 – 4.1)	0.3 (0.07 – 1.4)	1.3 × 10^–1^
	Primary	1,031 (51.4)			0.3 (0.04 – 3.0)	0.2 (0.04 – 0.8)	**2.6 × 10**^**–2**^
	NFE	233 (11.5)			0.6 (0.06 – 5.5)	0.3 (0.06 – 1.5)	1.4 × 10^–1^
**8**	**Occupation**						
	Artisanal (Ref)	1,272 (62.7)	15.634	**5.0 × 10**^**–3**^	1.0	1.0	Ref
	Civil Servant	122 (6.0)			*1.6 (0.4 – 5.7)	*2.7 (1.1 – 6.5)	**2.5 × 10**^**–2**^
	Causal Labour	206 (10.2)			*3.1 (0.9 – 10.3)	*3.4 (1.8 – 6.2)	**1.0 × 10**^**–4**^
	Unemployed	430 (21.2)			*2.4 (1.2 – 4.6)	*4.0 (2.7 – 5.9)	**1.0 × 10**^**–4**^
**9**	**Monthly income**						
	< 100	1,575 (77.6)	22.7198	**5.0 × 10**^**–4**^	-	-	-
	101–200	195 (9.6)			-	-	-
	201–300	132 (6.5)			-	-	-
	301–400	81 (4.0)			-	-	-
	≥ 401	47 (2.3)			-	-	-

Legend. number

*Most likelihood category

95%C.I 95%Confidence Interval, **Boldface** numbers indicate significant *p*-values, *n *frequency/count, *OR *Odds Ratio, Reference category of binomial regression, Good, Ref: Reference

As presented in (Table 4), binomial analysis revealed significant associations of occupation of respondents and knowledge of childhood vaccinations with good trust in childhood vaccinations (*p* > 0.05). The mean trust score was 95.16% (SD 16.52, range 0 – 100%).

**Table 4:** Binomial and multinomial logistic regression analyses of trust in childhood vaccinations with respondents' characteristics (*n* = 1,943)

		** Binomial regression**			**Multinomial Regression**		
**S/N**	Characteristic	*n* (%)	***χ***^***2***^	*p* – value	OR (95% C.I)	aOR (95% C.I)	*p*—value
**1**	**Age (in years)**						
	≤ 30 (Ref)	961 (49.5)	0.225	8.9 × 10^–1^	1.0	1.0	Ref
	31 – 40	841 (43.3)			0.9 (0.6 – 1.3)	0.9 (0.7 – 1.1)	1.6 × 10^–1^
	≥ 41	141 (7.3)			0.7 (0.4 – 1.3)	0.7 (0.5 – 1.1)	1.0 × 10^–1^
**2**	**# of Children in immunisation bracket**						
	01 child (Ref)	1,873 (96.4)	0.483	4.8 × 10^–1^	1.0	1.0	Ref
	≥ 02 Children	70 (3.6)			0.7 (0.3 – 1.5)	0.7 (0.5 – 1.1)	1.0 × 10^–1^
**3**	**Sex**						
	Female (Ref)	1,601 (82.4)	0.359	5.1 × 10^–1^	1.0	1.0	Ref
	Male	342 (17.6)			0.7 (0.4 – 1.3)	0.6 (0.5 – 0.8)	**1.3 × 10**^**–3**^
**4**	**Relationship**						
	Mother (Ref)	1,649 (84.9)	0.018	8.9 × 10^–1^	1.0	1.0	Ref
	Caregiver	294 (15.1)			*1.4 (0.7 – 2.5)	*1.5 (1.1 – 2.2)	**6.3 × 10**^**–3**^
**5**	**Religion**						
	Catholic	73 (3.8)	0.045	8.3 × 10^–1^	0.3 (0.4 – 2.9)	0.9 (0.6 – 1.3)	6.5 × 10^–1^
	Protestant (Ref)	1,870 (96.2)			1.0	1.0	Ref
**6**	**Marital status**						
	Not married	414 (21.3)	0.005	9.3 × 10^–1^	0.9 (0.6 – 1.4)	1.0 (0.8 – 1.2)	9.9 × 10^–1^
	Married (Ref)	1,529 (78.7)			1.0	1.0	Ref
**7**	**Educational status**						
	Tertiary (Ref)	65 (3.4)	4.906	1.8 × 10^–1^	1.0	1.0	Ref
	Secondary	655 (33.7)			*1.1 (0.4 – 2.6)	*1.2 (0.7 – 1.8)	4.5 × 10^–1^
	Primary	994 (51.2)			0.9 (0.4 – 2.3)	0.9 (0.6 – 1.5)	6.9 × 10^–1^
	NFE	229 (11.8)			*1.7 (0.6 – 5.2)	*1.9 (1.1 – 3.4)	**2.3 × 10**^**–2**^
**8**	**Occupation**						
	Artisanal (Ref)	1,252 (64.4)	10.403	**1.5 × 10**^**–2**^	1.0	1.0	Ref
	Civil Servant	107 (5.1)			0.4 (0.2 – 0.7)	0.5 (0.3 – 0.7)	**1.0 × 10**^**–4**^
	Causal Labour	186 (9.6)			0.5 (0.3 – 0.9)	0.7 (0.5 – 1.0)	**5.5 × 10**^**–2**^
	Unemployed	398 (20.5)			0.6 (0.4 – 1.0)	0.8 (0.6 – 0.9)	**1.2 × 10**^**–2**^
**9**	**Monthly income**						
	< 100	1,527 (78.6)	0.764	9.4 × 10^–1^	Ref	Ref	
	101–200	178 (9.2)			*1.1 (0.6 – 1.9)	0.9 (0.7 – 1.3)	7.9 × 10^–1^
	201–300	121 (6.2)			*1.3 (0.6 – 2.5)	*1.3 (0.9 – 1.9)	1.3 × 10^–1^
	301–400	73 (3.7)			*1.1 (0.5 – 2.4)	0.9 (0.6 – 1.4)	6.9 × 10^–1^
	≥ 401	44 (2.3)			*2.3 (0.6 – 8.9)	*2.3 (1.2 – 4.5)	**1.6 × 10**^**–2**^
**10**	**Knowledge**						
	Poor	79 (4.1)	9.408	**2.1 × 10**^**–3**^	*2.5 (1.5 – 4.5)	*2.6 (1.9 – 3.5)	**1.0 × 10**^**–4**^
	Good (Ref)	1,864 (95.9)			Ref	Ref	

Legend: number

*Most likelihood category

*95%C.I *95%Confidence Interval, **Boldface** numbers indicate significant *p* values, *OR *adjusted Odds Ratio, Reference Category of binomial regression: Very Poor, Ref: Reference

Of the 2,126 respondents, only 628 (29.5%, 95%C.I; 27.6 – 31.5) had both very good knowledge of – and trust in childhood vaccinations (Fig. 3). From MNLR analysis, the odds for trust in childhood vaccination was significantly higher amongst caregivers (*p* = 6.3 × 10^–3^, aOR; 1.5, 95%C.I; 1.1 – 2.2), respondents with no formal education (*p* = 2.3 × 10^–2^, aOR; 1.9, 95%C.I; 1.1 – 3.4) as well as those with secondary educational status (*p* = 4.5 × 10^–1^, aOR; 1.2, 95%C.I; 0.7 – 1.8), and those with a monthly income of more than $400 (*p* = 1.6 × 10^–2^, aOR; 2.3, 95%C.I; 1.2 – 4.5) as well as those with monthly income range of $201 – 300 (*p* = 1.3 × 10^–1^, aOR; 1.3, 95%C.I; 0.9 – 1.9) (Table 4).

Binomial analysis revealed no significant associations of demographic characteristics of respondents with both good knowledge of – and good trust in childhood vaccinations. From MNLR analysis, the odds for having both good knowledge of – and trust in childhood vaccination was significantly higher amongst caregivers (*p* = 4.0 × 10^–4^, aOR; 1.7, 95%C.I; 1.3 – 2.3), respondents with NFE status (*p* = 3.3 × 10^–2^, aOR; 1.7, 95%C.I; 1.0 – 3.0) as well as those with secondary educational status (*p* = 2.3 × 10^–1^, aOR; 1.3, 95%C.I; 0.8 – 2.0), persons with unemployed occupational (*p* = 2.0 × 10^–2^, aOR; 1.2, 95%C.I; 1.0 – 1.4), and average monthly income earners of ≥ $401 (*p* = 2.0 × 10^–4^, aOR; 3.5, 95%C.I; 1.8 – 6.8) as well as those between $201 – 300 vs $101 – 200 (*p* = 1.0 × 10^–4^, aOR; 2.1, 95%C.I; 1.4 – 2.9) vs (*p* = 2.3 × 10^–2^, aOR; 1.4, 95%C.I; 1.0 – 1.8) when compared with their counterparts (Table 5).

**Table 5:** Binomial and multinomial logistic regression analyses on knowledge of – and trust in childhood vaccination with respondents' characteristics (*n* = 1,864)

		** Binomial regression**			**Multinomial Regression**		
**S/N**	**Characteristic**	***n* (%)**	***χ***^***2***^	***p* – value**	**OR (95% C.I)**	**aOR (95% C.I)**	***p* – value**
**1**	**Age (in years)**						
	≤ 30 (Ref)	920 (49.4)	0.058	9.7 × 10^–1^	1.0	1.0	Ref
	31 – 40	808 (43.4)			1.0 (0.7 – 1.3)	0.9 (0.8 – 1.1)	5.5 × 10^–1^
	≥ 41	136 (7.3)			0.8 (0.5 – 1.4)	0.8 (0.6 – 1.1)	2.8 × 10^–1^
**2**	**# of Children in immunisation bracket**						
	01 child (Ref)	1,796 (96.4)	0.071	7.8 × 10^–1^	1.0	1.0	Ref
	≥ 02 Children	68 (3.6)			0.8 (0.4 – 1.6)	0.8 (0.6 – 1.2)	3.1 × 10^–1^
**3**	**Sex**						
	Female (Ref)	1,529 (82.0)	0.283	5.9 × 10^–1^	1.0	1.0	Ref
	Male	335 (18.0)			0.9 (0.6 – 1.5)	0.8 (0.6 – 1.0)	8.3 × 10^–2^
**4**	**Relationship**						
	Mother (Ref)	1,575 (84.5)	1.692	1.9 × 10^–1^	1.0	1.0	Ref
	Caregiver	289 (15.5)			*1.6 (0.9 – 2.7)	*1.7 (1.3 – 2.3)	**4.0 × 10**^**–4**^
**5**	**Religion**						
	Catholic	72 (3.8)	0.027	8.6 × 10^–1^	*1.1 (0.5 – 2.2)	1.0 (0.7 – 1.4)	9.9 × 10^–1^
	Protestant (Ref)	1,792 (96.2)			1.0	1.0	Ref
**6**	**Marital status**						
	Not married	401 (21.5)	0.459	4.9 × 10^–1^	*1.1 (0.8 – 1.5)	*1.2 (0.9 – 1.4)	7.2 × 10^–2^
	Married (Ref)	1,463 (78.5)			1.0	1.0	Ref
**7**	**Educational status**						
	Tertiary (Ref)	64 (3.4)	6.893	7.5 × 10^–2^	1.0	1.0	Ref
	Secondary	638 (34.2)			*1.3 (0.6 – 3.0)	*1.3 (0.8 – 2.0)	2.3 × 10^–1^
	Primary	941 (50.5)			0.9 (0.4 – 2.3)	0.9 (0.6 – 1.4)	5.8 × 10^–1^
	NFE	221 (11.9)			*1.8 (0.7 – 4.8)	*1.7 (1.0 – 3.0)	**3.3 × 10**^**–2**^
**8**	**Occupation**						
	Artisanal (Ref)	1,186 (63.6)	1.036	7.9 × 10^–1^	1.0	1.0	Ref
	Civil Servant	106 (5.7)			0.5 (0.3 – 0.8)	0.6 (0.4 – 0.8)	**4.9 × 10**^**–3**^
	Causal Labour	184 (9.9)			0.6 (0.4 – 1.1)	0.9 (0.6 – 1.2)	3.7 × 10^–1^
	Unemployed	388 (20.8)			1.0 (0.7 – 1.4)	*1.2 (1.0 – 1.4)	**2.4 × 10**^**–2**^
**9**	**Monthly income**						
	< 100	1,449 (77.7)	5.926	2.0 × 10^–1^	Ref	Ref	
	101–200	177 (9.5)			*1.6 (0.9 – 2.7)	*1.4 (1.0 – 1.8)	**2.3 × 10**^**–2**^
	201–300	121 (6.5)			*2.1 (1.1 – 4.1)	*2.1 (1.4 – 2.9)	**1.0 × 10**^**–4**^
	301–400	73 (3.9)			*1.8 (0.8 – 4.1)	*1.5 (0.9 – 2.2)	6.9 × 10^–2^
	≥ 401	44 (2.5)			*3.8 (1.0 – 14.2)	*3.5 (1.8 – 6.8)	**2.0 × 10**^**–4**^

Legend: number, *Most likelihood category, 95%C.I; 95%Confidence Interval, **Boldface** numbers indicate significant *p*-values, *n*; frequency/count, *OR :*Odds Ratio, aOR: adjusted Odds Ratio, Reference category of binomial regression: Good, Ref: Reference

## Discussion

The Rwandan Ministry of Health identified elements that would enhance immunisation uptake and programmed requirements in collaboration with local and international non-governmental organisations (NGOs) in order to meet sector development goals; goal 4 and reduce the under-five mortality rate by two-thirds by 2030 [53].

Our study adds to the description of the behaviour of mothers and caregivers towards childhood vaccination in Rwanda, where there is a gap in literature. This study revealed that; 91.8% of respondents obtained information about vaccines from health care workers. Majority of the mothers and caregivers had knowledge of childhood vaccinations, trust in childhood vaccinations, and had knowledge of – and trust in childhood vaccination.

### Sources of information

The adoption of vaccinations in Africa and around the world is impacted by misleading information about immunisations, lack of understanding and confidence in vaccination, and other factors.

Our study shows that the major source of information on vaccines and vaccination was provided by healthcare providers, followed by the mass media and social media. This was in line with previous studies in Greece, Cyprus and Switzerland, where parents rely on paediatricians for information concerning childhood vaccination [6, 60, 61], but differed from studies in the Netherlands, Philippines, Palestine, and Guinea where parents mostly explored the internet, as well as rely on traditional authorities for information about childhood vaccinations [15, 62–64]. In our study, 11.9% of respondents had information on vaccines and vaccinations from the social media; this was very low compared to the 22.07 – 49.5% reported elsewhere in Saudi Arabia and Palestine [15, 65]. Our finding was also different from that of a study conducted in rural communities to enhance vaccine confidence, wherein trusted messengers had lots of challenges communicating the importance of COVID-19 vaccine importance to the population [17]. In our study, there was 91.4% trust in childhood vaccination which was very high when compared with the 45.9% and 49.2% COVID-19 vaccine confidence reported amongst Filipinos and Malaysians respectively [66]. In other studies, parents considered factors like access to information, interpersonal communication, misinformation, and community norms for childhood vaccination [31, 67–69].

### Knowledge of mothers/caregivers regarding childhood vaccination

The 95.5% proportion of respondents with good knowledge of childhood vaccination in this study was high compared with the 86% reported among Saudi Arabian mothers [65], and very high compared with the 27 – 37.2% reported by other authors among Indonesian, Egyptian and Ethiopian parents [53, 70–72], and similar to the 91.7 – 94.4% reported among mothers in Italy and Greece [73, 74]. In another study on mothers' knowledge towards children's vaccination in Lebanon, good knowledge depended on physician’s communication [1]. The average knowledge score of 73.18% of our study was lower than the 86% reported in Saudi Arabia [65], and very high when compared with the 7.36 -13.6% reported amongst Cypriots and Malaysian parents [6, 75]. In terms of median scores, we had 75% (15/20), which was higher than the 11 reported in Greece [61]. The differences in the different studies might be due to differences in study designs.

In this study, 92% of respondents agreed (46.4% strongly agreed and 45.6% agreed) that vaccines are effective in the prevention of VPD; this was less than the 81.2 – 95.9% reported in Malaysia and Southeast Asia [76, 77]. A majority, 97.8% of agreed (49.3% strongly agreed and 48.5% agreed) that vaccines are beneficial and safe to the community; this was higher than the 41.8% reported in Palestine [15].

Furthermore, our study, found a significant association between good knowledge of childhood vaccination and sex, education, and occupation. Mother’s education, has been widely reported as an important determinant of knowledge in childhood vaccination in Ethiopia, Greece, Malaysia, and Palestine [15, 18, 61, 71]. On the other hand, research studies in the rural communities of the United States of America, showed that the factors affecting vaccine acceptance and knowledge were mistrust and misinformation as well as constantly changing health guidelines [17]..

### Trust of mothers/caregivers regarding childhood vaccination

In our study, 1,943 (91.4%) of the mothers/caregivers indicated that they had trust in childhood vaccination, which was higher than the 48.2—66% reported in other studies conducted in the Washington State and Saudi Arabia [78, 79], slightly higher than the 84% vaccine acceptance due to trust in six Southeast Asian countries [77] and lower than 91.6% reported amongst mothers in Greece [74]. Trust in childhood vaccination depended on the socio-demographic characteristics; sex, relationship with child(ren), educational status, occupation of parents/monthly income, as well as on the sources of vaccine information. This was different from the socio-demographic characteristics; sex, residence, educational status, occupational status, marital and family economic status as enumerated in a multi-national study in Southeast Asia [77]; vaccine convenience and doctor’s recommendations in Malaysia [76]. In other studies on the factors influencing parents' views on childhood vaccination, many complex factors; practices surrounding the illness condition, the people they interact with, politics, educational status, and access to vaccines [7, 74].

### Knowledge and trust of mothers/caregivers regarding childhood vaccination

Of the 2,030 and 1,943 who had knowledge of childhood vaccination and trust in childhood vaccinations, 1,864 (87.7%) had both knowledge of – and trust in childhood vaccinations. In similar studies elsewhere, good knowledge depended on parent to physician communication [1, 65].

Similar to our study, a studies in Spain and Lebanon revealed that knowledge of vaccination uptake was influenced by one's socioeconomic class, sex, and level of education [1, 80, 81]. However, a study in Lebanon also revealed that, a parent’s knowledge of childhood vaccination was influenced by monthly salary and the type of insurance [1].

Our study also revealed that 92% (45.6% agreed and 46.4% strongly agreed) of the respondents perceived that vaccines are effective. This was very high compared to the 78.8% reported for a global survey of 20 countries [26]. From our results, it was revealed that socio-demographic characteristics were associated with knowledge and trust in childhood vaccinations. This was contrary to the convenience, health provider’s advice, and cost of vaccine as reported in another study [26].

### Strengths and limitations

#### Strengths

Field data were obtained by field staff who had a mastery of the terrain. The quality of the data collected was assured through pretesting of questionnaires in a pilot study. The objective was to minimise bias as well as errors. The minimisation of bias was done by randomisation in the selection of Provinces, Districts, Sectors, Cells, and Villages. Further, bias was minimised by the use of a large sample size.

#### Limitations

This was a cross-sectional study, representing the snapshot of the population within the study period. Thus, we cannot infer causal relationships between mothers' knowledge of – trust in childhood vaccination with their demographic characteristics. Data were collected by convenient sampling of parents through anonymous self-reporting via door-to-door, and thus there is a possibility of double selection bias, response bias, and recall bias. Such biases can also affect some of the responses and subsequently the results of the study. Another significant limitation was representativeness as a higher proportion of the sampled respondents were undereducated, having acquired less than the secondary level of education.

## Conclusion

The majority of parents in Rwanda have good knowledge of – and trust in childhood vaccination. Knowledge and trust were good amongst mothers. This study indicates that there was an association between knowledge of childhood vaccination and relationship with the child(ren), education and occupation of the parent; it also indicated that there was no association between trust in childhood vaccination and parents' characteristics.

### Supplementary Information

**Additional file 1: S1 Appendix.** Survey Questionnaire.

**Additional file 2: S2 Appendix.** Supplementary File.

## Data Availability

All relevant data that support the conclusion of this study are included in the article. This is a field study rather than a clinical study.No data registration is required

## Abbreviations

95%C.I: 95% Confidence Interval
$$\overline{x}$$: Mean
Aor: Adjusted Odds Ratio
EPI: Expanded Programme on Immunisation
MNLR: Multinomial Logistic Regression
OR: Odds Ratio
SD: Standard deviation
VA: Vaccine acceptance
VH: Vaccine hesitancy
VPD(s): Vaccine preventable disease(s)
WHO: World Health Organisation
χ^2^;: Chi square
