# Supplementary material for: Knowledge and trust of mothers regarding childhood vaccination in Rwanda
Source: BMC Public Health. 2024 Apr 17;24:1067. doi: 10.1186/s12889-024-18547-1 (PMC11022416; doi:10.1186/s12889-024-18547-1)
Supplement: Supplementary file 1 — Additional file 1: S1 Appendix. Survey Questionnaire. [file 12889_2024_18547_MOESM1_ESM.docx]

Supplementary Sheet1

Assessment of childhood vaccination programme in rural and urban setting

**Survey form/ Questionnaire**

| S/N | **Section A- Parental demographic Information** |  |
| --- | --- | --- |
| 1. | Age of the parent |  |
|  | 1. 30 and below 2. 31-40 3. 41-50 4. 51-60 5. 61 and above |  |
| 2. | Sex of resident. |  |
|  | 1. Male 2. Female |  |
| 3. | Marital status. |  |
|  | 1. Single 2. Married 3. Widowed 4. Separated (Single parent) |  |
| 4. | Relationship with the child |  |
|  | 1. Mother 2. Caregiver 3. Father 4. Relative |  |
| 5. | Religion of the respondent |  |
|  | 1. Christian 2. Islam 3. Traditional religion 4. Any other please specify…………………… |  |
| 6. | Level of education |  |
|  | 1. None 2. Primary level 3. Secondary level 4. Tertiary level or higher |  |
| 7. | Occupation of respondent |  |
|  | 1. Civil servant 2. Businessman 3. Famer 4. Unemployed |  |
| 8. | Monthly Income of the respondent |  |
|  | 1. Less than $100 (less than 98,000RWF) 2. $101-$200(98980-196000 RWF) 3. $201-$300 (196980- 294000RWF) 4. $301-$400 (294980- 392000RWF) 5. $401-$500 (392980- 490000RWF) 6. More than $500 (490000RWF) |  |
| 9. | Where do you get information about child vaccination? |  |
|  | 1. Health workers (Nurses/Doctors) 2. Traditional leaders 3. Religious leaders 4. Mass Media (Radio/TV) 5. Social media (Facebook, Twitter, WhatsApp) 6. Friends, co-workers, or neighbours 7. Others |  |
|  | **Section B: Information about Child Immunization Status** |  |
| 10. | Do you have children in the immunization age bracket? |  |
|  | 1. Yes 2. No |  |
| 11. | How many children do you have in the immunization age bracket?  *For each child, please answer the questions from 12 to 20*: |  |
| 12. | Sex of the child |  |
|  | 1. Male 2. Female |  |
| 13. | Age of the child |  |
|  | 1. 0-6 Months 2. 7-12 Months 3. 13-18 Months 4. 19-24 Months |  |
| 14. | Did your child receive routine immunization |  |
|  | 1. Yes 2. No |  |
| 15. | What is the child’s immunization status |  |
|  | 1. Never immunized. 2. Partially immunized 3. Fully immunized |  |
| 16. | What evidence do you have to question 16 above? |  |
|  | 1. I remember what immunization dosages the child was given. 2. I have the immunization card. 3. I remember and have the immunization card. 4. I remember and the child has a scar. 5. I remember, I have the immunization card and the child has a scar |  |
| 17. | Have you ever delayed having your child immunized? |  |
|  | 1. Yes 2. No 3. I don’t know |  |
| 18. | Have you ever decided not to have your child vaccinated for reasons other than allergies or illness? |  |
|  | 1. Yes 2. No 3. I don’t know |  |
| 19. | **If yes** what was the reason for not having the child vaccinated? |  |
|  | 1. Was told vaccines can kill my child. 2. There was no one able to take the child to hospital. 3. I lost track of immunization dates and missed the doze. 4. Other specify……………………………. |  |
| 20. | On a scale of 1-3, how sure are you that following the recommended immunization schedule is good for your child. |  |
|  | 1. Sure 2. Not Sure 3. I don’t know   *Repeat the questions 12-20 for another child(ren)* |  |
|  | **Factors influencing child completion of vaccination doses** |  |
| 21. | What is the most important reason for not taking your child(ren) for vaccinations. |  |
|  | 1. My child was not born in hospital. 2. I cannot access to a health centre. 3. I do not believe vaccines are good for my child. 4. My religion does not permit me to immunize my children. 5. My culture does not permit me to immunize my children. 6. Others: please specify_______ |  |
| 22. | What is the most important reason for not completing vaccine doses? |  |
|  | 1. The child looks health and I feel there is no need to continue the doses. 2. I do not think these vaccines are good for my child. 3. One of my neighbour’s children got some health complications and they said it was due to the vaccine shots. 4. My religion does not permit me to immunize my children. 5. My culture does not permit me to immunize my children. 6. Others: please specify______ |  |
|  | **Parent’s knowledge, attitudes, and behaviour on child immunization.** |  |
| 23. | Does your religion support immunization of children? |  |
|  | 1. Yes 2. No 3. Neither supports nor opposes 4. I don’t know |  |
| 24. | Does your culture support immunization of children? |  |
|  | 1. Yes 2. No 3. Neither supports nor opposes 4. I don’t know |  |
| 25. | Does lack of funds ever prevented you from immunization of your child |  |
|  | 1. Yes 2. No 3. I don’t know |  |
| 26. | Does the message you hear or receive from anti-vaccine groups or propaganda affect your confidence of vaccination of your children? |  |
|  | 1. Yes 2. No 3. I don’t know |  |
| 27. | Do you trust the information you receive about vaccination of your children? |  |
|  | 1. Yes 2. No 3. I don’t know |  |
| 28. | All things considered, how much do you trust government and the public health agencies promote childhood vaccination? |  |
|  | 1. Trust. 2. Do not trust. 3. I do not know. |  |
| 29. | Are you able to openly discuss about vaccination of your child with your doctor or health care provider? |  |
|  | 1. Yes 2. No 3. I don’t know |  |
|  | **Vaccine attitude perception**  **How much do you agree with the each of the following statement on vaccinations?** |  |
| 30. | Vaccines are important for my health |  |
|  | 1. Agree. 2. Strongly agree. 3. Disagree 4. Strongly disagree. 5. Neither agree nor disagree. |  |
| 31. | Vaccines are effective |  |
|  | 1. Agree. 2. Strongly agree. 3. Disagree 4. Strongly disagree. 5. Neither agree nor disagree. |  |
| 32. | Getting myself vaccinated is important for the health of others in my community. |  |
|  | 1. Agree. 2. Strongly agree. 3. Disagree 4. Strongly disagree. 5. Neither agree nor disagree. |  |
| 33. | All vaccines offered by the government programs in my community are beneficial |  |
|  | 1. Agree. 2. Strongly agree. 3. Disagree 4. Strongly disagree. 5. Neither agree nor disagree. |  |
| 34. | New vaccines are more risky than older vaccines |  |
|  | 1. Agree. 2. Strongly agree. 3. Disagree 4. Strongly disagree. 5. Neither agree nor disagree. |  |
| 35. | I trust the information I receive about vaccines |  |
|  | 1. Agree. 2. Strongly agree. 3. Disagree 4. Strongly disagree. 5. Neither agree nor disagree. |  |
| 36. | Getting vaccinated is a good way to protect myself from disease. |  |
|  | 1. Agree. 2. Strongly agree. 3. Disagree 4. Strongly disagree. 5. Neither agree nor disagree. |  |
| 37. | I am able to openly discuss my concerns about vaccines with my doctor. |  |
|  | 1. Agree. 2. Strongly agree. 3. Disagree 4. Strongly disagree. 5. Neither agree nor disagree. |  |
| 38 | I am concerned about serious adverse effects of vaccines. |  |
|  | 1. Agree. 2. Strongly agree. 3. Disagree 4. Strongly disagree. 5. Neither agree nor disagree. |  |
| 39. | People do not need vaccines for diseases that are not common anymore. |  |
|  | 1. Agree. 2. Strongly agree. 3. Disagree 4. Strongly disagree. 5. Neither agree nor disagree. |  |
| 40. | People get more vaccinations than necessary. |  |
|  | 1. Agree. 2. Strongly agree. 3. Disagree 4. Strongly disagree. 5. Neither agree nor disagree. |  |
| 41. | It is better to develop immunity by getting sick than to get vaccinated. |  |
|  | 1. Agree. 2. Strongly agree. 3. Disagree 4. Strongly disagree. 5. Neither agree nor disagree. |  |
|  | **COVID-19 Vaccine: How much do you agree with each of the following statement on vaccinations?** |  |
| 42. | COVID-19 vaccines are important for my health. |  |
|  | 1. Agree. 2. Strongly agree. 3. Disagree 4. Strongly disagree. 5. Neither agree nor disagree. |  |
| 43. | COVID-19 vaccines are effective. |  |
|  | 1. Agree. 2. Strongly agree. 3. Disagree 4. Strongly disagree. 5. Neither agree nor disagree. |  |
| 44. | Getting myself vaccinated with a COVID¬19 vaccine is important for the health of others in my community. |  |
|  | 1. Agree. 2. Strongly agree. 3. Disagree 4. Strongly disagree. 5. Neither agree nor disagree. |  |
| 45. | All COVID-19 vaccines offered by the government program in my community are beneficial. |  |
|  | 1. Agree. 2. Strongly agree. 3. Disagree 4. Strongly disagree. 5. Neither agree nor disagree. |  |
| 46. | COVID-19 vaccines from other countries are more risky than vaccines from my country. |  |
|  | 1. Agree. 2. Strongly agree. 3. Disagree 4. Strongly disagree. 5. Neither agree nor disagree. |  |
| 47. | I trust the information I receive about COVID-19 vaccines. |  |
|  | 1. Agree. 2. Strongly agree. 3. Disagree 4. Strongly disagree. 5. Neither agree nor disagree. |  |
| 48. | Getting vaccinated against COVID-19 is a good way to protect myself from this disease. |  |
|  | 1. Agree. 2. Strongly agree. 3. Disagree 4. Strongly disagree. 5. Neither agree nor disagree. |  |
| 49. | Generally, I do what my doctor or health care provider recommends about COVID-19 vaccines for myself. |  |
|  | 1. Agree. 2. Strongly agree. 3. Disagree 4. Strongly disagree. 5. Neither agree nor disagree. |  |
| 50. | I am concerned about serious adverse effects of COVID-19 vaccines |  |
|  | 1. Agree. 2. Strongly agree. 3. Disagree 4. Strongly disagree. 5. Neither agree nor disagree. |  |
| 51. | I do not need to get a COVID-19 vaccine if it’s not a pandemic anymore. |  |
|  | 1. Agree. 2. Strongly agree. 3. Disagree 4. Strongly disagree. 5. Neither agree nor disagree. |  |
| 52. | I am concerned that COVID-19 vaccines might not prevent the disease. |  |
|  | 1. Agree. 2. Strongly agree. 3. Disagree 4. Strongly disagree. 5. Neither agree nor disagree. |  |
| 53. | I am concerned that COVID-19 vaccines might not be safe. |  |
|  | 1. Agree. 2. Strongly agree. 3. Disagree 4. Strongly disagree. 5. Neither agree nor disagree. |  |
| 54. | If a COVID­19 vaccine becomes available for me, I will get it |  |
|  | 1= Yes  2=No |  |
| 55. | In the last 3 months, have you tested for Covid19 positive. |  |
|  | 1= Yes  2=No |  |
|  | **Impact of COVID 19 on mothers continued routine Immunization** |  |
| 56. | Have you ever received or heard negative information about Covid19 vaccine? |  |
|  | 1= Yes  2=No |  |
| 57. | Do you trust negative information on Covid19? |  |
|  | 1= Trust  2=No Trust |  |
| 58. | Has COVID 19 affected your routine immunization? |  |
|  | 1= Yes  2=No |  |
| 59. | How did the Covid19 pandemic SOPs affect your routine immunization **during** the lockdown? |  |
|  | 1. There were no means of transport, so my child missed some vaccine doses. 2. My child could not access adequate treatment and has developed some complications. 3. The pandemic did not affect routine immunization |  |
|  | **Recommendations to improve completion of child immunization doses** |  |
| 60. | What measures do you think can help motivate parents to complete their child vaccine doses? |  |
|  | 1. Seek appropriate information about immunization from health workers. 2. Keep records to help them track the immunization calendars for their children. 3. Seek information about immunization to make informed decisions about immunization. 4. Do routine health check-up for their children to ascertain the impact of vaccines on the children. 5. Do their best to avail meet the nutrition needs of their children. |  |
| 61. | What measures should government adopt to improve vaccination programs? |  |
|  | 1. Put in place government programs to distribute vaccines in hard-to-reach areas with mobile clinics. 2. Put in places reliable sources of information about child vaccination. 3. Increase the number of health facilities in the rural areas. 4. Others specify |  |
| 62. | What measures should NGOs and Development partners adopt to improve vaccination programs. |  |
|  | 1. Increase funding to meet the vaccination needs for the hard to reach. Areas. 2. Package information on immunization to increase awareness. 3. Put in place check for false or unreliable information concerning immunization. 4. Conduct routine trainings. |  |

**End of the questionnaire**
