# Supplementary material for: Knowledge and trust of mothers regarding childhood vaccination in Rwanda
Source: BMC Public Health. 2024 Apr 17;24:1067. doi: 10.1186/s12889-024-18547-1 (PMC11022416; doi:10.1186/s12889-024-18547-1)
Supplement: Supplementary file 2 — Additional file 2: S2 Appendix. Supplementary File. [file 12889_2024_18547_MOESM2_ESM.docx]

**Supplementary Table 1: Association of Respondents Characteristics and Districts in Rwanda**

|  | District | | | | | |  |  |
| --- | --- | --- | --- | --- | --- | --- | --- | --- |
| General Characteristic | **Ngoma** | **Ngororero** | **Nyagatare** | **Nyamagabe** | **Nyarugenge** | **Total (%)** | **χ^2^** | ***p*-value** |
| Age groups (in years) |  |  |  |  |  |  |  |  |
| ≤ 30 | 223 | 165 | 279 | 157 | 226 | 1,050 (49.4) | 46.738 | **1.7x10^-7^** |
| 31 – 40 | 182 | 187 | 198 | 147 | 206 | 920 (43.3) |  |  |
| > 41 | 51 | 38 | 17 | 9 | 41 | 156 (7.3) |  |  |
| Mean age ($\overline{x}$± SD) |  |  |  |  |  | 31.03 ± 6.58 |  |  |
| Sex |  |  |  |  |  |  |  |  |
| Male | 100 | 126 | 78 | 6 | 68 | 378 (17.8) | 120.643 | **3.9x10^-25^** |
| Female | 356 | 264 | 416 | 307 | 405 | 1,748 (82.2) |  |  |
| Relation to child |  |  |  |  |  |  |  |  |
| Mother | 390 | 279 | 417 | 293 | 426 | 1,805 (84.9) | 82.908 | **4.2x10^-17^** |
| Caregiver | 66 | 111 | 77 | 20 | 47 | 321 (15.1) |  |  |
| Religion |  |  |  |  |  |  |  |  |
| Catholic/ Ntaho | 8 | 3 | 12 | 25 | 33 | 81 (3.8) | 45.513 | **3.1x10^-9^** |
| Protestant | 448 | 387 | 482 | 288 | 440 | 2,045 (96.2) |  |  |
| Marital status |  |  |  |  |  |  |  |  |
| Not married | 95 | 58 | 104 | 86 | 109 | 452 (21.3) | 17.694 | **1.4x10^-3^** |
| Married | 361 | 332 | 390 | 227 | 364 | 1,674 (78.7) |  |  |
| Education |  |  |  |  |  |  |  |  |
| No Formal Education | 70 | 23 | 58 | 65 | 25 | 241 (11.3) | 398 | **1.0x10^-77^** |
| Primary | 322 | 268 | 265 | 111 | 127 | 1,093 (51.4) |  |  |
| Secondary | 59 | 95 | 158 | 126 | 281 | 719 (33.8) |  |  |
| Tertiary | 5 | 4 | 13 | 11 | 40 | 73 (3.4) |  |  |
| Occupation |  |  |  |  |  |  |  |  |
| Artisan | 434 | 303 | 282 | 278 | 54 | 1,351 (63.5) | 970.009 | **5.2x10^-200^** |
| Casual labour | 6 | 40 | 45 | 28 | 90 | 209 (9.8) |  |  |
| Civil Servant | 3 | 22 | 51 | 1 | 48 | 125 (5.9) |  |  |
| Unemployed | 13 | 25 | 116 | 6 | 281 | 441 (20.7) |  |  |
| Number of Children in immunization bracket |  |  |  |  |  |  |  |  |
| 1 | 439 | 378 | 465 | 309 | 456 | 2,047 (96.3) | 12.074 | **1.7x10^-2^** |
| ≥ 02 | 17 | 12 | 29 | 4 | 17 | 79 (3.7) |  |  |
| Mean children ($\overline{x}$± SD) |  |  |  |  |  | 1.04 ± 0.24 |  |  |
| Monthly Income ($) |  |  |  |  |  |  |  |  |
| < 100 | 317 | 386 | 423 | 289 | 254 | 1,669 (78.5) | 426.033 | **1.3x10^-80^** |
| 101-200 | 51 | 4 | 54 | 24 | 64 | 197 (9.3) |  |  |
| 201-300 | 45 | 0 | 11 | 0 | 76 | 132 (6.2) |  |  |
| 301-400 | 34 | 0 | 1 | 0 | 46 | 81 (3.8) |  |  |
| > 400 | 9 | 0 | 5 | 0 | 33 | 47 (2.2) |  |  |
| Total | **456** | **390** | **494** | **313** | **473** | **2,126** |  |  |
| National population[34] | 404,048 | 367,955 | 653,861 | 371,501 | 16,665 | **1,814,030** |  |  |
